# Staff Elections at King Edward VII.'s Hospital, Cardiff

**Published:** 1913-03-08

**Authors:** 


### Staff Elections at King Edward VII.'s Hospital, Cardiff.

Alterations of the rules of the hospital were unanimously adopted at the annual meeting of governors of King Edward VII.'s Hospital, Cardiff, last week, presided over by the Lord Mayor of Cardiff (Alderman Morgan Thomas, J.P.). Their effect is to transfer from the board of management to an election committee the authority to appoint the members of the honorary medical staff. The governors are to be heartily congratulated, a correspondent writes, for this much needed reform, and the alterations have been made at the instance of the board of management. This body is recognised as being unsuitable for the purpose, its members numbering nearly 400 persons, many of whom attend the meetings very irregularly, or perhaps not at all. The new election committee is to be composed of the chairman of the board of management and the chairman of the five sub-committees, the honorary treasurer of the hospital, the principal and the dean of the Medical Faculty of the University College of South Wales and Monmouthshire, together with twelve members of the board of management, to be elected by the board for this special purpose. Colonel Bruce Vaughan announces that the invested capital of the hospital has now reached £120,000, and in response to their appeal to complete the Welsh Medical School, now closely allied to the hospital, a sum of £12,700 has been promised by Mr. W. James Thomas, of Ynyshir, to provide a physiological laboratory. Cardiff would no longer be content with a secondary place with regard to her medical school, and it would not be too much to hope, with the present wave of prosperity at the Docks, that some of their merchant princes would follow the excellent lead already given and complete the sum of £50,000 required for the new laboratories of the medical school. Continuing, Colonel Bruce Vaughan stated they were more immediately concerned with the need for opening the fifty remaining beds in the new wing. There were over 800 sufferers waiting to be treated, but denied admission because the additional income of £3,500 was not available. In supporting this vote, Mr. T. J. Hughes, the chairman of the Welsh National Insurance Commissioners, stated that under the Insurance Act there was a specific sum which might be ear-marked for research work. The Welsh Commissioners had an eminent Welsh bacteriologist, who was conducting research work in the rural parts of Wales into the cause of phthisis, and they hoped to have a valuable report soon. As to the future, it would be a matter of first-class importance, as it might be possible to ear-mark some considerable portion of the sum in relation to the medical school in order that a man of European reputation might be appointed to take charge of the research side in that great work. The Lord Mayor and the Lady Mayoress propose to initiate in Cardiff what is known as a "Queen Alexandra Day" on behalf of the hospital.

